# Sensor Fusion of Motion-Based Sign Language Interpretation with Deep Learning

**DOI:** 10.3390/s20216256

**Published:** 2020-11-02

**Authors:** Boon Giin Lee, Teak-Wei Chong, Wan-Young Chung

**Affiliations:** 1 School of Computer Science, The University of Nottingham Ningbo China, Ningbo 315100, China; boon-giin.lee@nottingham.edu.cn; 2 Department of Electronic Engineering, Keimyung University, Daegu 42601, Korea; chongteakwei@gmail.com; 3 Department of Electronic Engineering, Pukyong National University, Busan 48513, Korea

**Keywords:** deep learning, human-computer interaction, motion sensor, sensor fusion, sign language recognition, wearable computing

## Abstract

Sign language was designed to allow hearing-impaired people to interact with others. Nonetheless, knowledge of sign language is uncommon in society, which leads to a communication barrier with the hearing-impaired community. Many studies of sign language recognition utilizing computer vision (CV) have been conducted worldwide to reduce such barriers. However, this approach is restricted by the visual angle and highly affected by environmental factors. In addition, CV usually involves the use of machine learning, which requires collaboration of a team of experts and utilization of high-cost hardware utilities; this increases the application cost in real-world situations. Thus, this study aims to design and implement a smart wearable American Sign Language (ASL) interpretation system using deep learning, which applies sensor fusion that “fuses” six inertial measurement units (IMUs). The IMUs are attached to all fingertips and the back of the hand to recognize sign language gestures; thus, the proposed method is not restricted by the field of view. The study reveals that this model achieves an average recognition rate of 99.81% for dynamic ASL gestures. Moreover, the proposed ASL recognition system can be further integrated with ICT and IoT technology to provide a feasible solution to assist hearing-impaired people in communicating with others and improve their quality of life.

## 1. Introduction

Humans depend on many types of languages to convey messages and express themselves verbally and non-verbally. Nevertheless, hearing-impaired people are incapable of verbal communication with others. Because sign language expresses one’s meaning by relying upon the gestures of fingers, hands, arms, head, and body and on facial expressions [1], it has become the primary source of non-verbal communication for hearing-impaired people. In fact, sign languages are not standardized globally: some are one-handed but most are two-handed and/or a combination of both. The majority of sign languages require fingerspelling (especially for names and particular words, e.g., “OK”), but most words are represented by distinct combinations of gestures. Many studies on sign languages have been conducted over previous decades, including French Sign Language, Arabic Sign Language, Greek Sign Language, Chinese Sign Language (CSL), and Korean Sign Language [1].

**Figure 1:**
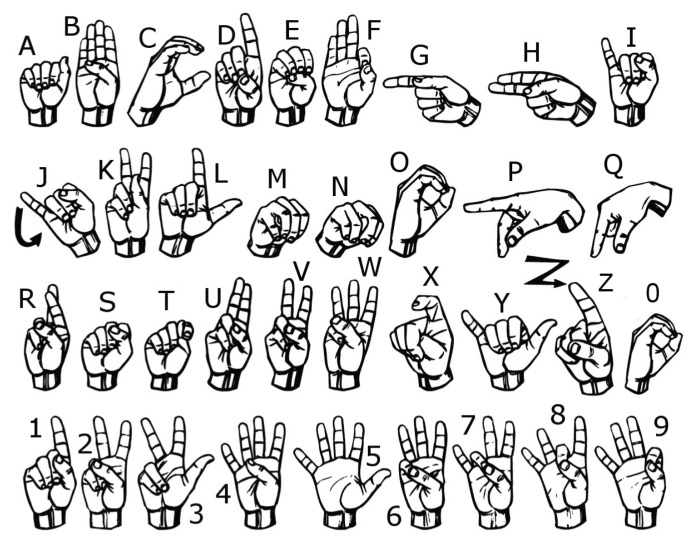
Fingerspelling in American Sign Language which represents 26 letters and 10 digits with different patterns of fingers and handshapes [4].

Specifically, a study by Ahmed et al. [2] revealed that American Sign Language (ASL) was the most frequently targeted sign language in linguistic research among all published papers from 2007 to 2017; that is, ASL was chosen as the principal sign language for research in most linguistic studies. Figure 1 shows the fingerspelling of ASL, which constitutes 36 gestures that represent 26 letters (A–Z) and 10 digits (0–9) with designated fingers and handshape gestures [3].

Most of the fingerspelling gestures of ASL are static and do not involve any movement, except for the letters J and Z that involve a simple hand movement. However, the majority of ASL signs are composed of dynamic gestures that consist of a series of basic or complex hand movements, combined with different handshapes and finger patterns to represent words or phrases. Spoken language can be broken down into simple sounds (phonemes). Likewise, sign language can also be broken down into segments of gestures that are composed of five basic components: handshape, orientation, articulation (position), movement, and facial-body expression [5,6].

Even though ASL gestures specifically correspond to English words and phrases, the representation of ASL gestures is not coded in the English language; thus, mastering the English language is not a prerequisite for learning ASL. Nonetheless, the grammar and structure of ASL sentences are more diverse and difficult than in the English language [7], which introduces challenges to mastering ASL in a short period of time. This eventually leads to the demotivation of society for learning ASL as their second language. The invisible communication barrier has become one of the many reasons that hearing-impaired people are isolated from society. In addition, there is a lack of accessibility in public facilities for hearing-impaired people, which are not designed specifically for them. It is also an issue for hearing-impaired people in interactions with staff in service sectors, such as a post office, in transportation, and at a bank counter. Moreover, there are cases when a hearing person loses their hearing and/or speaking ability either temporarily or permanently due to a severe accident. Hence, a sign language decoder (and encoder) application serves as an interpreter between the hearing-impaired community and public, with the objective of reducing the communication gap, and subsequently enhancing the quality of life of hearing-impaired people.

## 2. Related Works

With the dramatic development of human-computer interaction technology over previous decades, various studies have been conducted worldwide on the development of sign language recognition. Two approaches are widely adopted in sign language recognition: vision- and sensor-based. The vision-based approach utilizes an RGB camera and depth sensor and applies computer vision algorithms to analyze the hand gestures and body and facial expressions from images to recognize sign language [8]. On the other hand, the sensor-based approach derives finger and hand moving patterns (motion, position, and velocity) from multiple sensors that are attached to the user’s hands or body [9].

Cheok et al. [1] reported that images/videos for hand gesture analysis could be obtained from a single camera (i.e., webcam, video camera, and mobile device camera) or stereo-camera (i.e., multiple monocular cameras) using active or invasive techniques. Devices such as Microsoft Kinect, which recognizes the human body skeleton [10], and the Leap Motion Controller (LMC), which tracks hand movements [11], adopt an active technique that usually involves processing based on structured light projection. Meanwhile, the invasive technique detects the gestures of interest by tracking the moving patterns of preset markers attached to or worn by the users, for example, colored gloves (distinct colors to differentiate fingers). Elmezain et al. [12] proposed hand gesture recognition with a hidden Markov model (HMM) to detect “isolated” gestures (static hand position) and “meaningful” gestures (continuous motion of hand) for Arabic numbers with mean true recognition rates of 98.6% and 94.29%, respectively, using a Bumblebee stereo camera. The features of “meaningful” gestures were extracted from the hand motion trajectory through sequences of stereo color images. The study also proposed skin segmentation with a Gaussian mixture model over the YCbCr color space to overcome the issue of occlusion of the overlapping regions between the face and hands. On the other hand, Appenrodt et al. [8] presented a skin segmentation technique by comparing the hand motion trajectory from three different types of cameras (single color, stereo color, and thermal). The study reported that skin segmentation from images using a stereo camera with a depth sensor had the highest hand gesture recognition accuracy rate, where the depth information could segment the hand skin region (foreground) from the background. Likewise, Molchanov et al. [13] proposed a 3D-based convolutional neural network (CNN) multi-camera system that integrated a color camera, depth camera, and short-range radar sensor for the recognition of 10 dynamic hand gestures of drivers. The study indicated that CNN model classification with features extracted from color camera images showed the lowest true recognition rate of gestures at 60.1%, whereas CNN model classification using features extracted with “camera fusion” delivered the highest true recognition rate of gestures at 94.1%.

Apart from video cameras, several researchers adopted Kincet and LMC for detecting and tracking of hand gestures. Sykora et al. [14] classified 10 hand gestures with a detection rate of 82.8% using speeded up robust features based on depth information obtained from a Kinect depth sensor. Likewise, Chai et al. [15] presented a word- and sentence-based CSL recognition with a true accuracy rate of 96.32% by analyzing the 3D trajectory of the hand and body skeleton obtained from Kinect sensors. Meanwhile, Yang et al. [16] developed a 24 word-based sign language recognition system using Kinect 3D-information by applying a hierarchical conditional random field for gesture recognition. Chong et al. [4] developed an ASL (26 letters and 10 digits) recognition system with a true accuracy rate of 88.79% by applying a deep neural network, which outperformed a support vector machine (SVM) with a true accuracy rate of 72.79%. Nonetheless, environmental factors (i.e., lightning sensitivity, position, and detection range of camera and background noise) and occlusion (i.e., fingers or hands outside the vision sensor field of view) introduce challenges for computer vision approaches [17]. Alternatively, many researchers have begun to explore sensor-based approaches for sign language recognition.

In recent years, sensors have become capable of operating in low-power mode with the advancement of micro-electro-mechanical system (MEMS) technology, which is suitable for wearable computing solutions [18]. Flex sensors and inertial measurement units (IMUs) are the two general mechanical sensors used to track finger and hand movements [9]. Preetham et al. [19] developed two gloves with a total of 10 flex sensors (fabricated using low-cost electrostatic discharge material) attached on top of each pair of joints on each finger for gesture recognition. Similarly, Patil et al. [20] developed a single glove with five flex sensors attached on top of each finger that mapped the flexion of fingers into completed bend (finger closed), partial bend (finger half closed/opened), and straightened (finger opened). Complex sign languages usually comprise hand and finger movements; nonetheless, flex sensors are incapable of measuring finger orientation. In light of this issue, Das et al. [21] added a gyroscope sensor that was placed on the center of the back of the hand in addition to the five flex sensors. This increased the mean recognition rate to 86.67% for 20 dynamic gestures. Wang et al. [22] implemented a similar glove that consisted of a 3-axis accelerometer and five unidirectional flex sensors to recognize 50 CSL signs using a template-matching approach, achieving an accuracy rate of over 91%. Lee et al. [9] further improved the design by mounting two pressure sensors on top and left of the middle fingertip (right-handed), in addition to the five flex sensors and an IMU sensor. This improved the SVM classification accuracy rate significantly from 65.7% to 98.2% for 26 fingerspelled ASL signs; the pressure sensors resolved the issue in distinguishing the letters R, U, and V that exhibited the same flexion pattern but different movement. On the other hand, Mummadi et al. [23] utilized only five IMU sensors, one on each fingertip, to acquire pitch, raw, and yaw data of finger’s movement to recognize 24 static ASL letters (excluding letters J and Z) using random forest (RF) with an accuracy rate of 92.95%. Meanwhile, Lee et al. [24] designed finger gesture recognition using dynamic time warping (DTW) for smart home applications using three IMUs placed on the thumb, index finger, and back of the hand, respectively, and achieved a mean accuracy rate of 93.19%.

Moreover, Ong et al. [25] proposed a SIGMA system that applied vision-(using webcam) and sensor-based approaches (glove with nine resistive flex sensors which are placed on thumb (one sensor) and other fingers (two sensors each) along with 6-DOF IMUs) to recognize 26 letters, 10 digits, and 30 healthcare-related words from the Filipino Sign Language, e.g., “cough,” “doctor,” “physical exam,” “temperature,” and “allergy,” with a mean accuracy rate of 71.8% for letter and digit recognition and 80.6% for word recognition using the Viterbi algorithm. In general, both vision- and sensor-based approaches have their own merits and limitations. The vision-based approach is non-invasive but with a limited scope of field of view (region of interest), and image data are commonly affected by environmental noise. Nonetheless, the sensor-based approach offers high freedom of movement and mobility without the restriction of the limited scope of the field of view. However, the bulky design of wearable sensors is mostly a drawback and challenge in this approach. This study is aimed at designing novel and light wearable sensors to overcome the issue of bulky design by extending from the previous work [9] with 1. Introduction of word-based sign language, 2. Determination of features to differentiate words with similar pattern(s) (i.e., some words are similar in particular components but distinguishable in other components), and 3. Improvement of the classification algorithm with a deep neural network.

## 3. Materials and Methods

**Figure 2:**
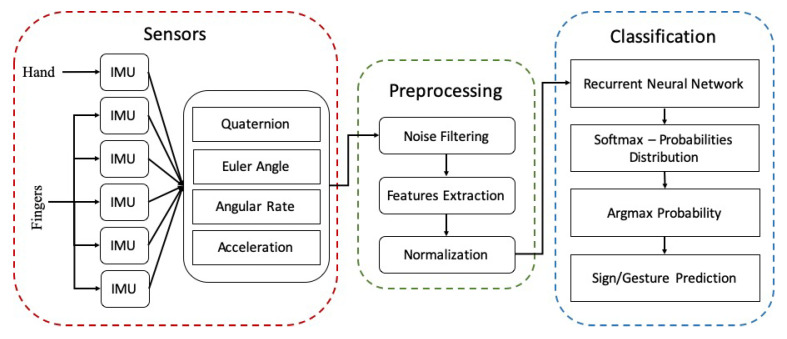
System overview of proposed smart wearable ASL recognition system.

The proposed smart wearable ASL gesture recognition system is composed of three modules: (1) sensing, (2) preprocessing, and (3) classification, as illustrated in Figure 2.

### 3.1. Data Collection

Twelve subjects were recruited from the university campus to participate in the experiments for collecting 27 word-based ASL gestures to form the dataset for model training and testing. Permission from the authorities to gather data from the hearing-impaired community requires significant delays and complex procedures, which limits the ability to quickly obtain gesture data from the community; however, collecting such data are planned for future work. The subjects were interviewed and selected candidates were requested to sign the consent form and information sheet. They were allowed to withdraw their participation at any time within the experimental period. Subjects that completed the experiments received an incentive of 100 USD for their efforts and contribution. Before the experiment, a short training video was provided for all the subjects with additional support from laboratory researchers with a 3-month period of learning experience in ASL. Each subject was requested to practice ASL gestures in a one-to-one training session (2 hours) with researchers to ensure that the subject fully understood and was familiarized with the gestures before the experiment. Each subject was also requested to wear the self-designed wearable device on their right hand for gesture data collection. A similar format of videos was displayed on the monitor screen as a reference while the subjects were performing the ASL gestures in random order with a duration of 10 s to 15 s for each gesture. Research assistants were monitoring on-site for safety purposes. Collected gestures were recorded under the supervision of the laboratory technician to ensure that the appropriate data was received. In addition, with the agreement from subjects, a webcam was placed on top of the monitor to record the entire experiment with subjects in the field of view for future reference. Ethical approval was obtained from the university’s research ethics panel (approval code: I01190900043).

**Figure 3:**
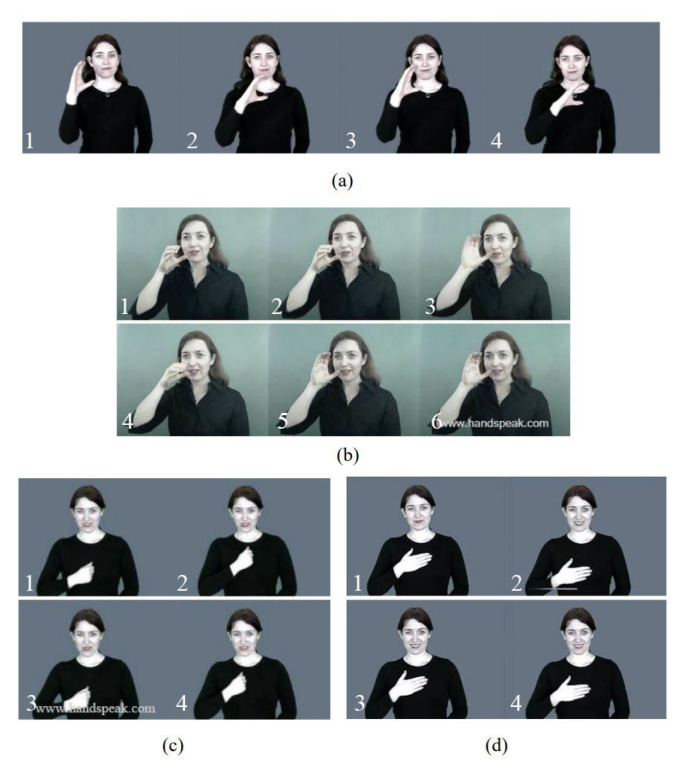
A series of images to illustrate the gestures that formed the ASL words (**a**) “search”, (**b**) “drink”, (**c**) “sorry”, and (**d**) “please” [27].

A total of 38,451 samples were collected, corresponding to 27 word-based ASL gestures. Word-based ASL is composed of five elements: (1) signation (SIG), (2) designator (DEZ), (3) orientation (ORI), (4) location (TAB), and (5) facial-body expression [26] that form the representation or signature of the word. SIG indicates the movements or actions of the hand palm, DEZ signifies the handshape patterns of the hand and fingers, ORI refers to the physical positions and directions of the hand palm, and TAB denotes the specific places or locations that the hand occupies. Table 1 lists the chosen ASL words used in this study, where certain words share the same component(s) but differ in other component(s). For instance, the word “search” has the same DEZ as the word “drink” but different SIG and TAB [6]; the words “sorry” and “please” have the same ORI but different DEZ [27] (see Figure 3).

### 3.2. Sensing Module

**Figure 4:**
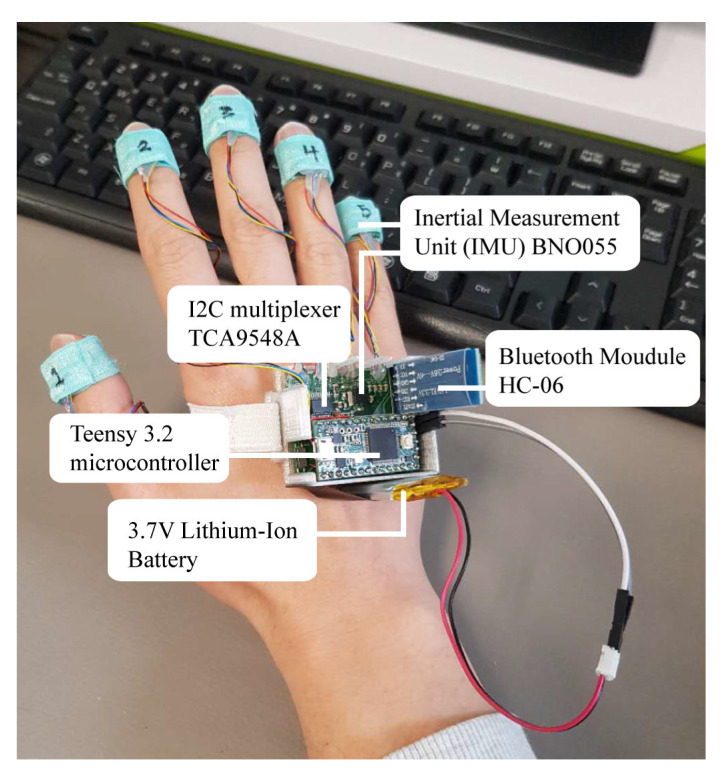
Proposed smart wearable ASL gesture recognition system.

The sensing module consists of six IMUs, one placed on top of each fingertip and the back of the hand, as depicted in Figure 4. The initial design in this study was to utilize a 9-DOF absolute orientation IMU module [28] (composed of accelerometer, gyroscope, and magnetometer), manufactured by Adafruit [29], but the size of the IMU module board was too large to be adopted as a wearable sensor solution (bulky design). Thus, a customized motion module was redesigned with a BNO055 IMU sensor chip to resolve this issue, which successfully reduced the overall weight by 30% and board size by 77%.

IMUs are wire connected to a Teensy 3.2 microcontroller (MCU) [30], which is equipped with a 72 MHz Cortex-M4 ARM processor, 256 KB flash memory, and 64 KB random-access memory (RAM) with a low operating voltage between 3.3V and 5V. This small-scale MCU could be overclocked to operate at 96 MHz and provide six times faster processing and 16 times more flash memory capacity than Arduino UNO [31]. In addition, two TCA9548 [32] multiplexers are employed as communication mediums between the MCU and all 6 IMUs as the Teensy MCU only had two I2C ports, which were insufficient to connect all the IMUs. Each multiplexer operates at low voltage between 1.65V to 5.5V and consists of four pairs of serial data (SDA) and serial clock (SCL) pins, which are capable of connecting up to 4 IMUs. The sensor data are further digitized and transmitted to a terminal via the HC-06 Bluetooth low energy (BLE 4.0) module [33], which is operated at a low voltage power between 3.3V and 5V, functioning at 2.4 GHz industrial, scientific, and medical (ISM) band. Table 2 summarizes the components in the sensing module with their respective specifications.

### 3.3. Data Preprocessing

Each IMU sensor delivers outputs of acceleration (ACC, m/s^2^, 100 Hz) from the accelerometer, angular rate (AGR, deg/s^2^, 100 Hz) from the gyroscope, and magnetic field (MGF, μT, 20 Hz) from the magnetometer in 3-axis non-fusion mode. The sensor fusion algorithm produces a stable orientation by “fusing” calibrated tri-axis MEMS accelerometer, gyroscope, and magnetometer and outputs in either a 3-axis Euler angle (ELA, 100 Hz) based on 360 deg. sphere or 4-points quaternion data (QTR, 100 Hz) with a high-speed ARM Cortex-MO processor [29]. In fact, ELA and QTR are two different orientation representations. ELAs are limited by a phenomenon called “gimbal lock” due to similarities between two phenomena, which prevents them from measuring orientation when the pitch angle approaches ± 90 degrees. Thus, QTRs are often used [34] and are included as features in this study for comparison. Nonetheless, the non-fusion MGF data do not show any obvious significance in describing gesture patterns [23]; thus, they were excluded from the analysis in this study.

Statistical methods: mean (μ) and standard deviation (σ) are computed from the raw sensor data (ACC, AGR, ELA, and QTR) as features because the information in raw sensor data are not suitable as input features, when movements within a short time interval are involved. σ is commonly introduced to describe the pattern of spread from the average distribution of a list of elements [35]. A low σ value of raw sensor data of a window size indicates no or nearly static movement (no gesture), whereas a high σ value signifies the occurrence of large movements (gestures). The μ value is computed to observe the average distribution of a specified window-size raw sensor data for each axis. The σ and μ computations are illustrated in Equations (1) and (2):

(1) $$\sigma =\sqrt{\frac{1}{N}\left(\sum_{i=1}^{N}{\left(x_{i}-\mu \right)}^{2}\right)}$$

(2) $$\mu =\frac{1}{N}\left(\sum_{i=1}^{N}{\left(x_{i}\right)}^{2}\right)$$

Here, *N* and *x* represent the total number of data instances of a window size and sensor data, respectively. The features *S* are organized as $S^{i}$, where i=1…6 represents the index of the IMU on the hand at the position of the back of the hand, thumb (fingertip), index fingertip, middle fingertip, ring fingertip, and pinky fingertip in sequential order. The IMU data ($S^{i}$) of ACC, AGR, and ELA are composed of 3-tuple dimensions (*x*, *y*, and *z* axes), whereas QTR is composed of 4D vectors with values between –1 and 1. The data structures are as follows:

$$S=\{S^{i}\};i=1\ldots 6$$

$$S^{i}=\left\{ACC^{i},AGR^{i},ELA^{i},QTR^{i}\right\}$$

$$ACC^{i}=\left\{ACC_{\sigma }^{i},ACC_{\mu }^{i}\right\}$$

$$ACC_{\sigma }^{i}=\left\{ACC_{\sigma x}^{i},ACC_{\sigma y}^{i},ACC_{\sigma z}^{i}\right\},$$

$$QTR_{\sigma }^{i}=\left\{QTR_{\sigma a}^{i},QTR_{\sigma b}^{i},QTR_{\sigma c}^{i},QTR_{\sigma d}^{i}\right\},$$

Here, σ and μ are computed for ACC, AGR, and ELA in *x*, *y*, and *z* axes, whereas, for QTR in *a*, *b*, *c*, and *d* elements. The elements *b*, *c*, and *d* are the “vector part” of the quaternion on which rotation is performed, and element *a* is the “scalar part” that specifies the amount of rotation performed on the “vector part.” To further investigate the significance of each feature and implication of sensor fusion with respect to the performance of the classification model, the features are subdivided into 15 categories ($C_{i}$), where each feature includes both the σ and μ values:

- $C_{1}$: QTR
- $C_{2}$: ELA
- $C_{3}$: AGR
- $C_{4}$: ACC
- $C_{5}$: QTR + ELA + AGR + ACC
- $C_{6}$: QTR + ELA + AGR (ACC excluded)
- $C_{7}$: QTR + ELA + ACC (AGR excluded)
- $C_{8}$: QTR + AGR + ACC (ELA excluded)
- $C_{9}$: ELA + AGR + ACC (QTR excluded)
- $C_{10}$: QTR + ELA (AGR and ACC excluded)
- $C_{11}$: QTR + AGR (ELA and ACC excluded)
- $C_{12}$: QTR + ACC (ELA and AGR excluded)
- $C_{13}$: ELA + AGR (QTR and ACC excluded)
- $C_{14}$: ELA + ACC (QTR and AGR excluded)
- $C_{15}$: AGR + ACC (QTR and ELA excluded)

The categories 1 to 4 consist of only a single type of IMU data, and category 5 consists of all types of IMU data. Meanwhile, categories 6 to 9 contain the leave-one-out type of IMU data (one type of IMU data are excluded, e.g., $S^{i}=\left\{ACC^{i},AGR^{i},ELA^{i}\right\}$), whereas each category of 10 to 15 consists of two different types of IMU data in unique combinations (2 types of IMU data are excluded, e.g., $S^{i}=\left\{ACC^{i},AGR^{i}\right\}$). The aim of this categorization is to investigate and determine the group(s) of the highest informative IMU data in classifying the dynamic movement patterns of ASL-related gestures. In addition, all extracted data are normalized by scaling the data values into the range of [0, 1] to reduce the computational complexity and ensure data consistency from different subjects [4]. Finally, the dataset is organized in the vector form (Equation (3)):

(3) $${\left(x_{j}^{i},y\right)}^{t};y=1,2\ldots 27$$

Here, *x* and *y* denote *j*-th feature of *i*-th IMU and label, respectively, at time *t*, e.g., the dataset for category 1 contains a total of 37D features (σ and μ for each IMU in *x*, *y*, and *z* axes with total of 6 IMUs and a label):

$${\left(x^{i},y\right)}^{t}={\left(ACC^{i},y\right)}^{t};i=1\ldots 6$$

### 3.4. Classification Model

**Figure 5:**
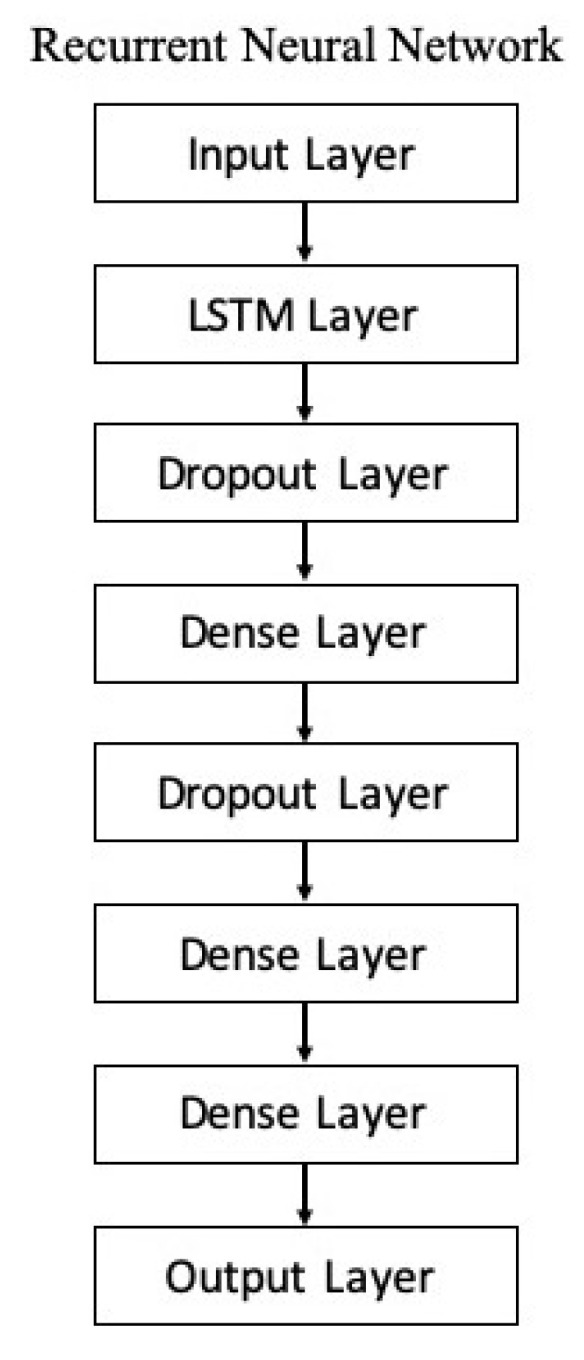
Proposed recurrent neural network model.

Figure 5 illustrates the design model of the recurrent neural network (RNN) proposed in this study to classify the 27 ASL gestures. The first layer is the *k*-dimensional input layer of input features followed by a long short-term memory (LSTM) layer. The LSTM was initially introduced by Hochreiter and Schmidhuber to resolve the problem of vanishing and exploding gradients in a network, and to enable the network to handle long-term dependencies [36]. The general LSTM consists of 3 gates to protect and control the cell state: 1) forget, 2) input, and 3) output. These gates are composed of a logistic sigmoid layer and a pointwise multiplication operation. The forget gate regulates the information optionally passing through the cell, which is the key idea of LSTM, controlled by sigmoid layer where the output value of 0 prevents anything from passing through, whereas output value of 1 allows 100% of information to pass through the gate. The sigmoid layer in input gate decides the state values to be updated by adding the new candidate values, which are generated by the hyperbolic tangent (tanh) function:

(4) $$f\left(x\right)=\frac{1-exp(-2x)}{1+exp(-2x)},$$

where the output of f(x) is further normalized to the value of [–1, 1] to accelerate the convergence process, which could prevent the cell memory from “blowing up” (exploding gradients problem) [37]. Next, a dropout layer (DR) is introduced to randomly drop a certain percentage of the neurons in the network during the network training process to prevent overfitting, that is, to preclude the neurons from co-adapting too frequently [38]. The next layer is a dense layer implemented with a rectified linear activation unit. Subsequently, a DR layer is added, followed by a dense layer. Another dense layer is added with 27 output neurons that correspond to the 27 ASL-related gestures (classes). The categorical distribution of each gesture, $p(y_{i})$, with respect to the IMU data, is computed by a softmax function

(5) $$p\left(y_{i}\right)=\frac{e^{y_{i}}}{\sum_{j=1}^{J}e^{y_{j}}};i=1\ldots 27$$

that normalizes the outputs to a probability distribution that consists of 27 gesture probabilities using sparse categorical cross-entropy as the loss function. The gesture is classified by the smooth approximation of the argument maximum function with the highest probability of all classes.

The RNN model is subjected to compilation using an adaptive moment estimation (Adam) algorithm as a gradient descent-based optimizer with a learning rate of 0.001 and decay rate of $5\times 10^{-5}$ (the first and second moment exponential decay rates are defined as 0.9 and 0.99, respectively). Two validation models are applied, hold-out and k-fold cross validation, to gauge the generalizability of the network during the model training process, to validate the learning performance, and to tune the hyperparameters of the network. The dataset is partitioned into 70%, 20%, and 10% for training, validation, and testing datasets, respectively, for the hold-out model, whereas 10-fold cross validation [39] is performed for the k-fold model.

## 4. Results and Discussion

First, the accuracy rate (AR, Equation 6) is applied to evaluate the performance of each RNN model, which is trained with 15 different categories of IMU data.

(6) $$AR=\frac{TP+TN}{TP+TN+FP+FN},$$

where *TP*, *TN*, *FP*, and *FN* are denoted as true positive, true negative, false positive, and false negative, respectively. Table 3 presents the accuracy rates of the trained RNN models using different combinations of IMU data as features with hold-out validation. The RNN models trained with at least two types of IMU data ($C_{5}$ to $C_{15}$) presented better performance (between 99.29% and 99.89%) than RNN models trained with only a single type of IMU data ($C_{1}$ to $C_{4}$). In the group of single type IMU data, the RNN model trained with only AGR showed the lowest accuracy rate of 97.89%, whereas the model trained with only QTR showed the highest accuracy rate of 98.51%. Meanwhile, among the RNN models trained with 2 types of IMU data, $C_{12}$ and $C_{11}$ achieved the highest and lowest mean accuracy rates of 99.66% and 99.29%, respectively. Likewise, among the RNN models trained with 3 types of IMU data, $C_{9}$ and $C_{6}$ achieved the highest and lowest mean accuracy rates of 99.73% and 99.48%, respectively. The RNN model trained with all types of IMU data, $C_{5}$, presents the highest overall accuracy rate of 99.83%.

On the other hand, Table 4 illustrates the accuracy rates of the trained RNN models using different types of IMU data as features with 10-fold cross validation. Likewise, the RNN model trained with all types of IMU data, $C_{5}$, presented the highest accuracy rate of 99.85%, slightly better than the RNN model with hold-out validation. The RNN model trained with only AGR ($C_{3}$) had the lowest performance with an accuracy rate of 99.56%. The performances are similar for the RNN model trained with 3 types of IMU data for hold-out and 10-fold cross validation. Overall, the accuracy rate of the trained RNN model presents a positive correlation with the number of types of IMU data used as input features for the RNN model, providing the credibility of sensor fusion to distinguish and predict the ASL-related gestures that involve dynamic movement patterns.

**Figure 6:**
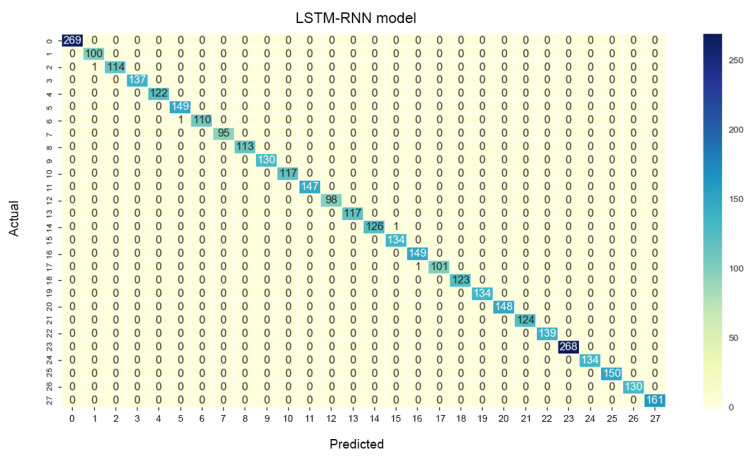
Confusion matrix of the trained RNN model.

Figure 6 depicts the confusion matrix of the RNN model trained with all types of IMU data ($C_{5}$; this RNN model is labeled as RNN_5_ model for the rest of the discussion). A “happy” gesture (class 2) was incorrectly predicted by the RNN_5_ as a “good” gesture (class 3) due to the same DEZ component but insufficiently distinguishing SIG and ORI components. Likewise, a “pretty” gesture (class 6) showed false prediction as an “understand” gesture (class 5); they have high similarity of TAB components but not DEZ, SIG, and ORI components. A “drink” gesture (class 17) was incorrectly predicted as a “please” gesture (class 16) as both gestures have the same starting position in the chest area. In addition, a “thank you” gesture (class 14) was incorrectly predicted as a “yes” gesture (labeled as class 15) due to a high percentage of similarity in TAB.

In summary, most of the false predictions are due to high similarity of TAB, and the trained RNN_5_ model showed low efficiency in distinguishing the articulation position (TAB) of those gestures. To further evaluate the performance of RNN_5_, two evaluation metrics were introduced: sensitivity (Se) and specificity (Sp). Se (Equation (7)) reflects the probability of the trained RNN_5_ to correctly identify the gestures with respect to all positive cases, whereas Sp (Equation (8)) reflects the probability of the trained RNN_5_ to reject the incorrectly identified gestures with respect to all negative cases.

(7) $$Se=\frac{TP}{TP+FN}$$

(8) $$Sp=\frac{TN}{TN+FP}$$

**Figure 7:**
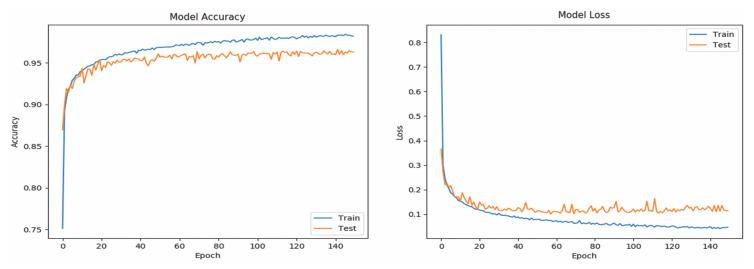
Performance of the trained RNN_5_ with high number of neurons evaluated with mean accuracy rate (**left**) and loss function (**right**).

**Figure 8:**
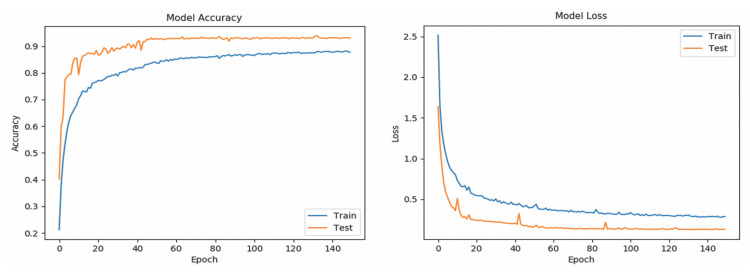
Performance of trained RNN_5_ with high DR value, evaluated with mean accuracy rate (**left**) and loss function (**right**).

**Figure 9:**
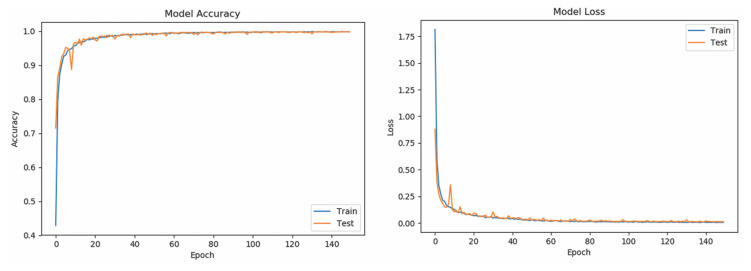
Performance of trained RNN_5_ with optimized DR value and number of neurons, evaluated with mean accuracy rate (**left**) and loss function (**right**).

**Figure 10:**
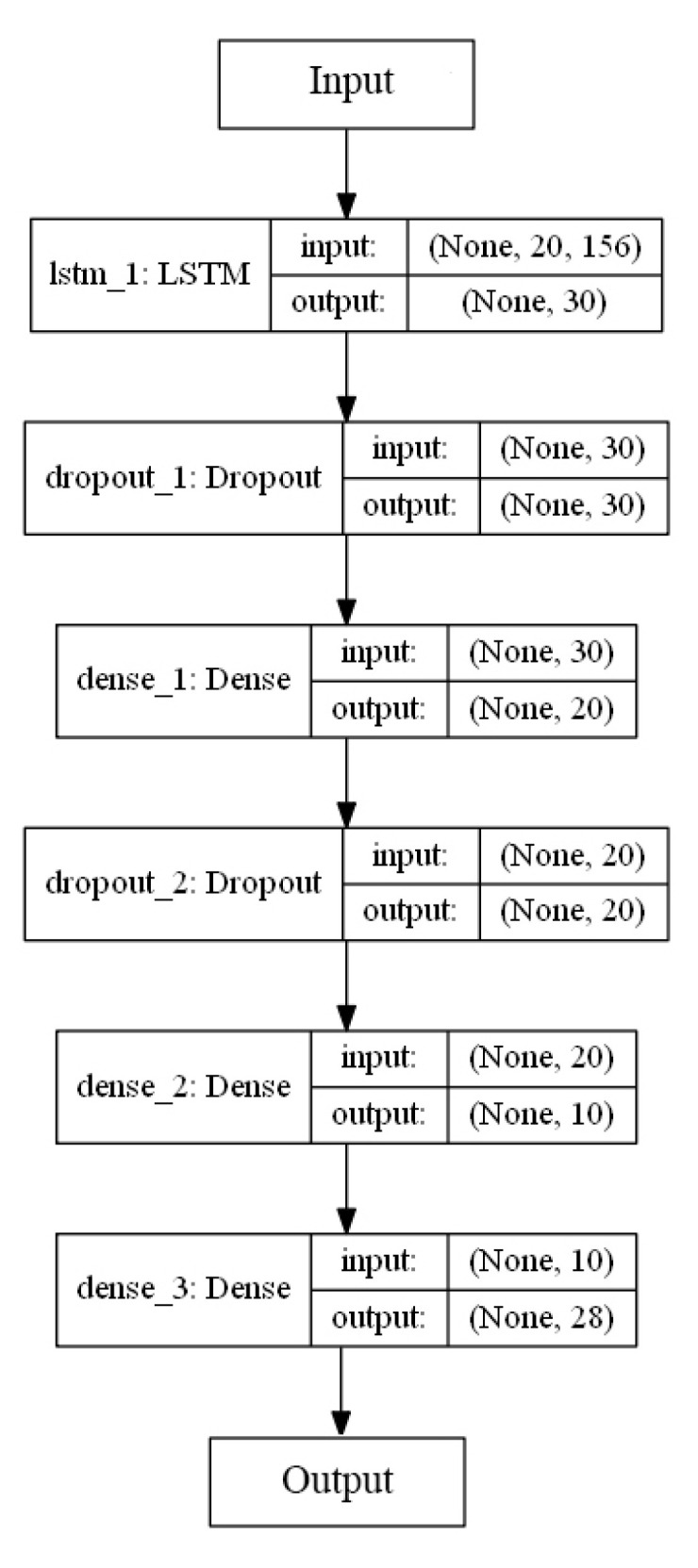
Optimized configuration of the RNN_5_ model.

Table 5 shows the Se and Sp of all classes (word-based ASL gestures) with promising results greater than 99%. The “drink” gesture has the lowest Se because there are some other gestures identified as “drink” gesture, similar to the cases of “pretty”, “happy”, and “thank you” gestures. Meanwhile, a “good” gesture is falsely identified (lower Sp than 100%), similarly to the “understand”, “yes”, and “please” gestures. In short, the results indicate that the RNN_5_ has a nearly perfect true negative rate, better than the true positive rate which is slightly lower. The performance of the RNN_5_ from an overall perspective was evaluated with the accuracy rate (left) and loss function (right), trained with a total of 150 epochs and a batch size of 20. Initially, the LSTM layer, dense layers (excluding the last dense layer), and DR layers (DR value of 0) were configured, each with a number of output neurons ≥40, and the performance was plotted, as shown in Figure 7. Overfitting occurred after the 20th epoch, with the testing accuracy rate remaining at approximately 95% and error rate of 0.1, indicating that RNN_5_ was overtrained (too high complexity). For further improvement, the DR value was set at 0.1 (randomly removing 10% of the neurons in the RNN_5_), and the performance is depicted in Figure 8. Even though the gaps in accuracy rate and error rate between the training and testing reduced as the epochs increased, the gaps were still significant, indicating that the DR value was high and the removal of meaningful neurons caused the performance to drop. Thus, manipulation or tuning of the DR value and number of neurons, stressed by Sheela et al. [40], were applied to overcome the overfitting issue for better performance. After several trials, the best performance was achieved by RNN_5_ (see Figure 9) with the configuration of 30 output neurons in LSTM, 30 output neurons for the first DR layer (DR value of 0.02), 20 output neurons for the first dense layer, 20 output neurons for the second DR layer (DR value of 0.01), and 10 output neurons for the second dense layer, as illustrated in Figure 10.

Finally, Table 6 illustrates the comparison of our proposed classification algorithm with conventional classification algorithms conducted by other researchers: hidden Markov model (HMM), support vector machine (SVM), random forest (RF), and dynamic time warping (DTW). Wang et al. [22] utilized an HMM classifier for 50 Chinese Sign Language signs and achieved 91% recognition accuracy. Meanwhile, Lee et al. [9] presented a model with an accuracy of 98.2% for 26 fingerspelling ASL gestures using an SVM classifier. Nevertheless, our proposed study presented the highest accuracy of 99.81% using the RNN-LSTM classifier for the recognition of 27 dynamic word-based ASL signs.

## 5. Conclusions

This paper presented the design and implementation of a wearable solution for word-based ASL interpretation by analyzing the movement patterns of fingers and hands based on motion data from IMU sensors. The RNN model with the LSTM layer was tuned to deliver the best performance for classifying 27 word-based ASL gestures with a mean accuracy rate of over 99%. The experimental results reveal that the four components of fingers and hand gestures (SIG, DEZ, ORI, and TAB) are represented by the sensor fusion of QTR, ELA, AGR, and ACC data, which serve as indicators for complex gestures, specifically for gestures that involve high dynamic movements. Even though the current study only considered single-handed word-based ASL, the preliminary results are promising for further study. Future work includes extension to two-handed word-based ASL and potentially to sentence-based ASL (and other sign languages) as well as incorporating an automated network hyperparameter fine-tuning algorithm for network optimization. This research could be widely adopted in fields such as the healthcare sector, for example, to enable severely injured patients (vocal injury) to communicate with the medical personnel in situations where verbal communication is restricted or limited.

## Figures and Tables

**Table 1:** A list of chosen ASL words indicated by their similarities.

Words	SIG	DEZ	ORI	TAB
”Good”-“Happy”		X		
”Happy”-“Smell”	X	X	X	
”Sorry”-“Please”	X		X	X
”Hungry”-“Drink”-“Search”		X		
”Pretty”-“Sleep”	X	X		X
”There”-“Me/I”-“You”-“Hearing”		X		
”Hello”-“Bye”		X	X	
”Thank You”-“Good”		X	X	X
”Yes”-“Sorry”		X		
”Eat”-“Water”	X			X
”Look”-“Vegetable”		X		
”Onion”-“Apple”	X	X	X	

**Table 2:** Components in a sensing module.

Components	Specification
	Tri-axial 16 bits gyroscope
IMU	Tri-axial 16 bits accelerometer
	Geomagnetic sensor
	Operating voltage: 2.4V to 3.6V
	Operating voltage: 3.3V to 5V
Teensy 3.2 MCU	Processor: Cortex-M4 72 MHz (96 MHz)
	Flash memory: 256 KB
	RAM: 64 KB
	I2C: 2 ports
	Operating voltage: 1.65V to 5.5V
TCA29548A multiplexer	Clock frequency: 0 to 400 kHz
	I2C: 3 ADDR pins, 8 buses (4 SDA/SCL)
	Operating voltage: 3.3V to 5V
BLE 4.0 HC-06	Frequency: 2.4 GHz ISM
	Transmission range: 10 m

**Table 3:** Accuracy rates of the trained RNN models using different categories of IMU data with hold-out validation.

Category	Features	AR (%)
$C_{1}$	QTR	98.51
$C_{2}$	ELA	98.44
$C_{3}$	AGR	97.89
$C_{4}$	ACC	98.45
$C_{5}$	QTR + ELA + AGR + ACC	99.83
$C_{6}$	QTR + ELA + AGR	99.48
$C_{7}$	QTR + ELA + ACC	99.64
$C_{8}$	QTR + AGR + ACC	99.68
$C_{9}$	ELA + AGR + ACC	99.73
$C_{10}$	QTR + ELA	99.46
$C_{11}$	QTR + AGR	99.29
$C_{12}$	QTR + ACC	99.66
$C_{13}$	ELA + AGR	99.39
$C_{14}$	ELA + ACC	99.59
$C_{15}$	AGR + ACC	99.56
	Average	99.67

**Table 4:** Accuracy rates of the trained RNN models using different categories of IMU data with 10-fold cross validation.

Category	Features	AR (%)
$C_{1}$	QTR	99.65
$C_{2}$	ELA	99.70
$C_{3}$	AGR	99.56
$C_{4}$	ACC	99.66
$C_{5}$	QTR + ELA + AGR + ACC	99.85
$C_{6}$	QTR + ELA + AGR	99.84
$C_{7}$	QTR + ELA + ACC	99.84
$C_{8}$	QTR + AGR + ACC	99.82
$C_{9}$	ELA + AGR + ACC	99.82
$C_{10}$	QTR + ELA	99.83
$C_{11}$	QTR + AGR	99.83
$C_{12}$	QTR + ACC	99.82
$C_{13}$	ELA + AGR	99.78
$C_{14}$	ELA + ACC	99.82
$C_{15}$	AGR + ACC	99.79
	Average	99.67

**Table 5:** Se and Sp of the trained RNN_5_ model.

Class	Se (%)	Sp (%)	Class	Se (%)	Sp (%)
None/Invalid	100	100	“Thank You”	99.21	100
”Good”	100	99.97	“Yes”	100	99.97
”Happy”	99.13	100	“Please”	100	99.97
”Sorry”	100	100	“Drink”	99.02	100
”Hungry”	100	100	“Eat”	100	100
”Understand”	100	99.97	“Look”	100	100
”Pretty”	99.10	100	“Sleep”	100	100
”Smell”	100	100	“Hearing”	100	100
”There”	100	100	“Water”	100	100
”You”	100	100	“Rice”	100	100
”Me/I”	100	100	“Search”	100	100
”OK”	100	100	“Onion”	100	100
”Hello”	100	100	“Apple”	100	100
”Bye”	100	100	“Vegetable”	100	100

**Table 6:** Comparison of sign language recognition sensor methods.

Reference	Sign Language	Sensor	Algorithm	AR (%)
Wang et al. [22]	50 CSL	3-axis ACC	HMM	91.00
		5 flex sensors		
Lee et al. [9]	26 fingerspelling ASL	1 IMU and 5 flex sensors	SVM	98.20
Mummadi et al. [23]	24 static ASL	5 IMU	RF	92.95
Lee et al. [24]	6 hand gestures	3 IMU sensors	DTW	93.19
Proposed	27 word-based ASL	6 IMU sensors	RNN-LSTM	99.81
